# Supplementary material for: Identification of photoperiod-induced specific miRNAs in the adrenal glands of Sunite sheep (Ovis aries)
Source: Front Vet Sci. 2022 Jul 22;9:888207. doi: 10.3389/fvets.2022.888207 (PMC9354845; doi:10.3389/fvets.2022.888207)
Supplement: Supplementary file 1 [file Data_Sheet_1.ZIP › Supplementary file/miRDeep2 file.zip!/miRDeep2 file/Know_Structure/SPLP42A2/oar-mir-382.pdf]

miRBase precursor : oar-mir-382
Total read count : 5852
oar-miR-382-5p read count : 2539
oar-miR-382-3p read count : 3300
remaining reads : 13

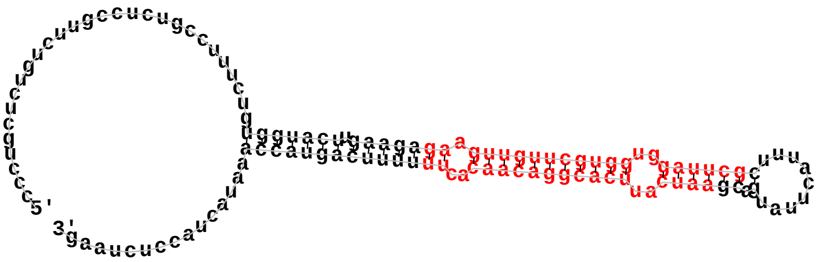

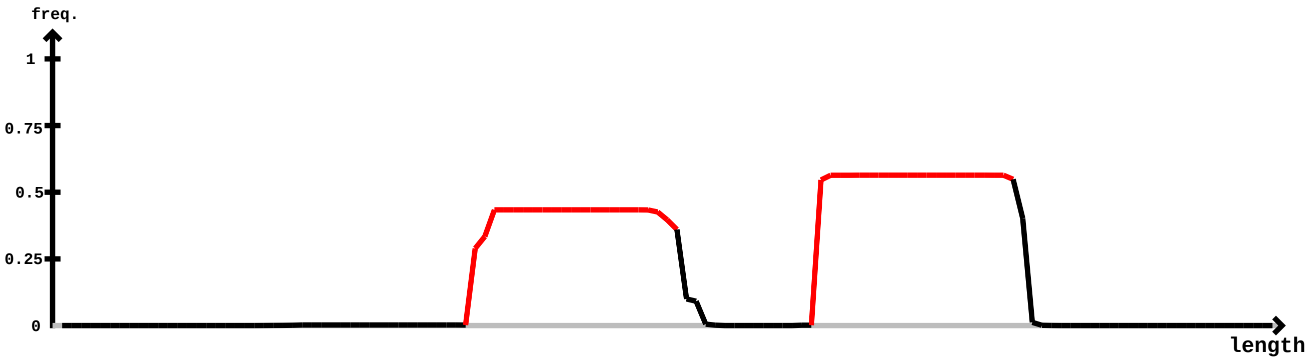


```
                                                                                           oar-miR-382-3p
                                             oar-miR-382-5p
5'- cccugcucugucuugccucugccuuucugugguacuugaagagaaguuguucgugguggauucgcuuuacuuaugacgaaucauucacggacaacacuuuuuucaguaccaaauacuaccucuaag  -3'    exp
    ....((........)).............(((((((.(((((((.(((((((((((..(((((((.........).))))))..)))))))))))..))))))))))))))...............   reads   mm
    ....................gccuuucugugguacuugaaga....................................................................................   1       0
    .....................ccuuucugugguacuugaaga....................................................................................   2       0
    ......................cuuucugugguacuugaaga....................................................................................   1       0
    .......................uuucugugguacuugaag.....................................................................................   1       0
    .......................uuucugugguacuugaaga....................................................................................   2       0
    ........................uucugugguacuugaaA.....................................................................................   1       1
    ........................uucugugguacuugaaga....................................................................................   4       0
    ........................uucugugguacuugaagag...................................................................................   1       0
    ..........................................gaaguuguucguggugga..................................................................   2       0
    ..........................................gaaguuguucgugguggaG.................................................................   2       1
    ..........................................gaaguuguucgugguggau.................................................................   41      0
    ..........................................gaaguuguucgugguggauu................................................................   165     0
    ..........................................gaaguuguucgugguggauuc...............................................................   195     0
    ..........................................gaaguuguucgugguggauucg..............................................................   1271    0
    ..........................................gaaguuguucgugguggauucgc.............................................................   15      0
    ..........................................gaaguuguucgugguggauucgcu............................................................   1       0
    ...........................................aaguuguucgugguggau.................................................................   2       0
    ...........................................aaguuguucgugguggauu................................................................   8       0
    ...........................................aaguuguucgugguggauuc...............................................................   14      0
    ...........................................aaguuguucgugguggauucg..............................................................   233     0
    ...........................................aaguuguucgugguggauucgc.............................................................   3       0
    ...........................................aaguuguucgugguggauucgcu............................................................   2       0
    ............................................aguuguucgugguggauu................................................................   1       0
    ............................................aguuguucgugguggauuc...............................................................   1       0
    ............................................aguuguucgugguggauucg..............................................................   20      0
    ............................................aguuguucgugguggauucgc.............................................................   32      0
    ............................................aguuguucgugguggauucgcu............................................................   500     0
    ............................................aguuguucgugguggauucgcuu...........................................................   22      0
    ............................................aguuguucgugguggauucgcuuu..........................................................   9       0
    ............................................................................cgaaucauucacggacaacacu............................   5       0
    ............................................................................cgaaucauucacggacaacacuu...........................   2       0
    ............................................................................cgaaucauucacggacaacacuuu..........................   1       0
    ..............................................................................aaucauucacggacaaca..............................   1       0
    ..............................................................................aaucauucacggacaacacu............................   84      0
```

```
                                                                             oar-miR-382-3p
                                          oar-miR-382-5p
cccugcucugucuugccucugccuuucugugguacuugaagagaaguuguucgugguggauucgcuuuacuuaugacgaaucauucacggacaacacuuuuuucaguaccaaauacuaccucuaag

.............................................................................aaucauucacggacaacacuu...........................   852    0
.............................................................................aaucauucacggacaacacuuu..........................   2210   0
.............................................................................aaucauucacggacaacacuuuu.........................   40     0
..............................................................................aucauucacggacaacacuu...........................   3      0
..............................................................................aucauucacggacaacacuuu..........................   70     0
..............................................................................aucauucacggacaacacuuuu.........................   24     0
..............................................................................aucauucacggacaacacuuuuu........................   4      0
..............................................................................aucauucacggacaacacuuuuuu.......................   2      0
.................................................................................auucacggacaacacuu...........................   1      0
.................................................................................auucacggacaacacuuu..........................   1      0
```
